# Dose-escalation using intensity-modulated radiotherapy for prostate cancer - evaluation of quality of life with and without ^18^F-choline PET-CT detected simultaneous integrated boost

**DOI:** 10.1186/1748-717X-7-14

**Published:** 2012-01-30

**Authors:** Michael Pinkawa, Marc D Piroth, Richard Holy, Jens Klotz, Victoria Djukic, Nuria Escobar Corral, Mariana Caffaro, Oliver H Winz, Thomas Krohn, Felix M Mottaghy, Michael J Eble

**Affiliations:** 1 Department of Radiation Oncology, RWTH Aachen University, Pauwelsstrasse 30, 52072 Aachen, Germany; 2 Department of Nuclear Medicine, RWTH Aachen University, Pauwelsstrasse 30, 52072 Aachen, Germany

**Keywords:** prostate cancer, intensity-modulated radiotherapy, simultaneous integrated boost, choline PET, quality of life

## Abstract

**Background:**

In comparison to the conventional whole-prostate dose escalation, an integrated boost to the macroscopic malignant lesion might potentially improve tumor control rates without increasing toxicity. Quality of life after radiotherapy (RT) with vs. without ^18^F-choline PET-CT detected simultaneous integrated boost (SIB) was prospectively evaluated in this study.

**Methods:**

Whole body image acquisition in supine patient position followed 1 h after injection of 178-355MBq ^18^F-choline. SIB was defined by a tumor-to-background uptake value ratio > 2 (GTV_PET_). A dose of 76Gy was prescribed to the prostate (PTV_prostate_) in 2Gy fractions, with or without SIB up to 80Gy. Patients treated with (n = 46) vs. without (n = 21) SIB were surveyed prospectively before (A), at the last day of RT (B) and a median time of two (C) and 19 month (D) after RT to compare QoL changes applying a validated questionnaire (EPIC - expanded prostate cancer index composite).

**Results:**

With a median cut-off standard uptake value (SUV) of 3, a median GTV_PET _of 4.0 cm^3 ^and PTV_boost _(GTV_PET _with margins) of 17.3 cm^3 ^was defined. No significant differences were found for patients treated with vs. without SIB regarding urinary and bowel QoL changes at times B, C and D (mean differences ≤3 points for all comparisons). Significantly decreasing acute urinary and bowel score changes (mean changes > 5 points in comparison to baseline level at time A) were found for patients with and without SIB. However, long-term urinary and bowel QoL (time D) did not differ relative to baseline levels - with mean urinary and bowel function score changes < 3 points in both groups (median changes = 0 points). Only sexual function scores decreased significantly (> 5 points) at time D.

**Conclusions:**

Treatment planning with ^18^F-choline PET-CT allows a dose escalation to a macroscopic intraprostatic lesion without significantly increasing toxicity.

## Background

Increasing doses for prostate cancer have been shown to be associated with improved biochemical control rates in several prospective randomized studies [1]. However, rectal toxicity has also increased significantly. Rectal toxicity is known to be the dose-limiting toxicity in prostate cancer radiotherapy [2,3]. Focusing the dose escalation on the actual tumour has the potential to increase tumour control without increasing toxicity.

Local prostate cancer recurrence after primary radiotherapy (RT) usually originates in the location of the primary tumor. Cellini et al. found the origin of all local recurrences within the initial tumor volume, supporting the rationale of a selective dose escalation only to the intraprostatic lesion [4]. In view of an often multifocal microscopic spread of prostate cancer [5] - not possible to delineate with the presently available imaging modalities - the whole prostate has still to be covered by an effective dose.

Treatment was based on ^18^F-choline PET-CT (positron emission tomography and computed tomography acquired in same patient position) for patients in focus of this study. A simultaneous integrated boost (SIB) was delivered to the GTV_PET _(gross target volume, as defined in PET). Quality of life (QoL) changes were evaluated prospectively and compared to a patient group treated with the same concept in the same time interval - but without a SIB - to demonstrate an acceptable treatment tolerance and a rationale to continue this innovative strategy.

In contrast to grading scales, for example the commonly applied RTOG/EORTC (Radiation Therapy Oncology Group/European Organisation of Research and Treatment of Cancer) scale, QoL questionnaires have the advantage of revealing all grades of toxicity from the patient's perspective. Baseline problems can be taken into account accurately.

## Methods

### Treatment planning

This prospective study was based on consecutive patients who were treated due to localized T1-3N0M0 prostatic carcinoma in the years 2008-2009. PET-CT (Philips Gemini TF 16) with ^18^F-choline was performed in 46 patients for treatment planning. Image acquisition in supine patient position followed 1 h after injection of 178-355MBq ^18^F-choline. Images were reconstructed using the iterative LOR (line of response) algorithm (pixel size: 4 mm^3^). CT images (from PET-CT) with a slice thickness of 3 mm were used for treatment planning.

The CTV (prostate volume ± base of seminal vesicles), bladder and rectum (region from anal canal to the rectosigmoid flexure) were delineated by identifying the external contours (Philips Pinnacle^3 ^Version 8.0 m). GTV_PET _(intraprostatic lesion) was defined by a tumour-to-background choline uptake ratio > 2, based on the results of studies correlating choline PET results with histopathologic examinations [6,7]. We have identified an area of about 1 cm^2 ^within the prostate with the lowest activity and defined the threshold for GTV_PET _after multiplying the SUV_max _(maximum standard uptake value) in this area by 2 [8].

For the planning target volume (PTV), 8 mm lateral and anterior, 5 mm superior and inferior and 4 mm posterior margin was added. A uniform margin of 4 mm was added to the GTV_PET _to account for intrafraction target motion, with the exception of the posterior direction (3 mm). Intrafraction prostate displacements have been evaluated in our department recently using ultrasound based image-guided radiotherapy (IGRT) - the required margins were calculated to be ≤4 mm in all directions, with 85% of displacements within a margin of 3 mm [9]. Ultrasound based IGRT was applied before each fraction for patient positioning for all patients using the BAT^® ^SXi system (B-mode acquisition and targeting).

**Figure 1:**
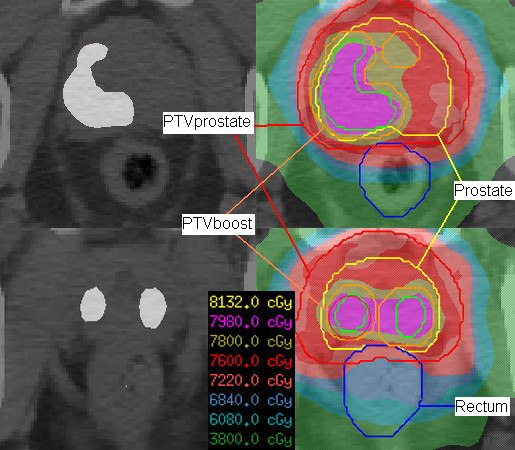
**Simultaneous integrated boost concept for two patients with a PET-CT slice (images on the left, PET signal demonstrates the malignant lesion above a fixed standard uptake value threshold) and the corresponding contours and isodose lines in a treatment plan (images on the right)**.

An inverse planning with a five field step-and-shoot intensity-modulated radiotherapy (IMRT) technique and 15MeV photons was used. The direct machine parameter optimization (DMPO) algorithm was applied for inverse planning with a 2 cm^2 ^minimum segment area, 5 minimum segment monitor units and a maximum number of 70 segments. The dose to the PTV was prescribed to a reference point (outside the volume of GTV_PET_). Treatment planning objectives are summarised in Table 1. Examples are shown in Figure 1.

**Table 1:** Treatment planning objectives

region of interest	type	objective
PTV_boost_	uniform dose = *prescription dose to GTV_PET_*	80Gy

PTV_boost_	dose homogeneity	+/-5%

CTV_prostate_	minimum dose	76Gy

PTV_prostate_	minimum dose	72Gy [95% of prescription dose]

PTV_prostate _- PTV_boost_	uniform dose = *prescription dose to PTV*	76Gy

Rectum	maximum dose	76Gy (constraint)

Rectum	maximum dose to 20% volume	70Gy

Rectum	maximum dose to 50% volume	50Gy

Bladder	maximum dose to 30% volume	70Gy

Bladder	maximum dose to 50% volume	55Gy

Quality of life and treatment plans for patients who were treated with SIB were compared with patients treated without SIB in the same period (n = 21) - boost volume has either have not been defined or ^18^F-choline PET-CT has not been performed due to logistic reasons. Tumour based criteria had no relevance for the selection to treat with or without SIB. With the exception of the SIB, the same treatment planning and treatment delivery technique was used for patients without SIB (daily IGRT and five field IMRT).

We have focused on the EUD (equivalent uniform dose) for the PTV, rectum and bladder and the NTCP (normal tissue complication probability) for the rectum and bladder.

$$EUD={\left(\frac{1}{N}\underset{i}{\sum }D_{i}^{a}\right)}^{\frac{1}{a}}$$

N = voxel number; D_i _= dose in the i'th voxel; a = tumour or normal tissue-specific parameter that describes the dose-volume effect; with a = -10 for prostate cancer and a = 6 for bladder and rectum in this study [8].

NTCP for grade 3 or worse rectum and bladder toxicity was computed applying the Lyman-Kutcher-Burman model with Emami parameters (rectum: n = 0.12, m = 0.15, median toxicity dose = 80Gy; bladder: n = 0.5, m = 0.11; median toxicity dose = 80Gy) [10,11].

### Quality of life evaluation

Patients were surveyed prospectively before (time A), at the last day (B), and a median time of two months (range 6 weeks-6 months) after (C) and 19 months (range 14-24 months) after (D) radiotherapy using a validated questionnaire, the Expanded Prostate Cancer Index Composite (EPIC) [12,13]. The questionnaire comprises 50 items concerning the urinary, bowel, sexual and hormonal domains for function and bothersomeness. The multi-item scale scores were transformed lineary to a 0-100 scale, with higher scores representing better QoL. According to data in the literature, mean QoL changes of below 5 points can be defined as clinically not significant, 5-10 as "little" changes, 10-20 as "moderate" changes and > 20 as "very much" changes [14]. Other authors use the "0.5 standard deviation of baseline value" to determine clinically relevant changes [14] - this is also reported in this study. A baseline questionnaire was required for inclusion in this study resulting in a total number of 46/21 (time A), 38/16 (time B), 44/21 (time C) and 44/20 (time D) questionnaires after treatment with/without SIB.

The questionnaire was handed over to the patients personally by one of the physicians at time A, B and C. Number of questionnaires was the lowest at the end of radiotherapy (time B) because this point in time was limited to a single day (last radiotherapy fraction) and there was no second opportunity to fill out a missed questionnaire. Patients presented in the department six to ten weeks after the end of treatment. Missed questionnaires in the acute phase (time C) and questionnaires one to two years after radiotherapy (time D) were sent to the patients with a return envelope. If a questionnaire was not returned within 4 weeks, patients were contacted by telephone and were urged to complete it. The median time after treatment and the respective ranges for the questionnaires C and D were the same for patients after treatment with and without SIB.

### Statistical analysis

Statistical analysis was performed using the IBM SPSS 19.0 software. The Wilcoxon's matched-pairs test was applied to determine longitudinal changes in specific subgroups of patients. To explore statistical volume or dose differences in treatment plans and QoL score differences between different subgroups at a specific time, the Mann-Whitney-U-test was used. Contingency table analysis with the chi-square test was performed to compare treatment groups with respect to categorical variables. All p-values reported are two-sided, p < 0.05 is considered significant.

## Results

Demographic and treatment plan-related data are presented in Table 2, showing well comparable (statistically not significantly different) distribution of prognostic factors and organ volumes for treatment planning. Median EUD values for patients treated with SIB were 2% higher in comparison to patients without SIB, with slightly higher NTCP for rectal toxicity. However, only the EUD for PTV_prostate _was significantly higher for patients treated with SIB (p > 0.05 for all other comparisons).

**Table 2:** Demographic and treatment plan-related data

	prostate with SIB (n = 46)	prostate only (n = 21)
patient age/years median (range)	72 (59-83)	71 (61-81)

PTV_prostate _/cc median (range)	142 (35-310)	163 (70-537)

PTV_boost _/cc median (range)	17 (4-46)	-

% PSA ≤10 ng/mL	74%	76%

% Gleason score ≤6	65%	71%

% T-stage ≤2a	78%	86%

% NHT	17%	19%

bladder volume/cc median (range)	237 (69-645)	225 (98-493)

rectum volume/cc median (range)	88 (36-235)	81 (48-252)

EUD PTV_prostate_*/Gy median (range)	76.7 (73.8-78.3)	76.0 (74.3-77.1)

EUD PTV_boost _/Gy median (range)	79.5 (76.6-80.5)	-

EUD bladder/Gy median (range)	55.6 (41.7-63.2)	54.6 (45.0-62.3)

EUD rectum/Gy median (range)	55.9 (47.1-62.4)	54.6 (40.1-60.6)

NTCP bladder/% median (range)	0 (0-0.6)	0 (0-0.4)

NTCP rectum/% median (range)	6.9 (2.2-14.7)	5.5 (1.5-11.2)

Abbreviations: PTV = planning treatment volume; PSA = prostate-specific antigen; NHT = neoadjuvant hormonal therapy; EUD = equivalent uniform dose; NTCP = normal tissue complication probability

*p < 0.01

With a median cut-off standard uptake value (SUV) of 3, a median GTV_PET _of 4.0 cm^3 ^and PTV_boost _(GTV_PET _with margins) of 17.3 cm^3 ^was defined. Malignant lesions were defined as single lesions in 31%, as two bilateral lesions in 26%, as central lesions extending into both lobes in 30% and as multiple (at least three) lesions in 13%.

Baseline QoL scores have not been found to be significantly different (Table 3) for patients treated with or without SIB. No significant differences were found regarding urinary, bowel and sexual QoL changes at times B, C and D (mean differences ≤3 points for all comparisons in urinary and bowel domains). Significantly decreasing acute urinary and bowel scores changes (clinically relevant: mean changes > 5 points in comparison to baseline level at time A; > 0.5 standard deviation of baseline value) were found for patients with and without SIB. However, long-term urinary and bowel QoL (time D) did not differ relative to baseline levels - with mean urinary and bowel function score changes < 3 points in both groups (median changes = 0 points).

**Table 3:** Mean function an bother scores before treatment and differences after treatment relative to baseline scores (quartiles in brackets; negative differences indicate a quality of life improvement)

		time A	0.5 SD	time A-time B	time A-time C	time A-time D	significant differences		
							
							A vs. B	A vs. C	A vs. D
urinary function score	prostate with SIB	94 (94;100;100)	6	12 (0;9;20)	5 (0;0;7)	1 (0;0;7)	+	+	-
	
	prostate only	89 (87;100;100)	9	9 (0;3;18)	3 (-3;0;8)	2 (0;0;8)	+	-	-

urinary bother score	prostate with SIB	82 (75;86;96)	8	17 (0;18;32)	2 (-10;0;11)	0 (-10;0;6)	+	-	-
	
	prostate only	79 (63;86;98)	10	20 (2;14;37)	6 (-4;4;20)	0 (-7;0;13)	+	-	-

bowel function score	prostate with SIB	92 (89;96;100)	5	10 (0;11;19)	3 (-3;0;7)	1 (-4;0;4)	+	-	-
	
	prostate only	90 (89;96;100)	6	14 (1;14;24)	3 (-4;4;9)	-1 (-6;0;7)	+	-	-

bowel bother score	prostate with SIB	92 (89;100;100)	6	12 (0;14;21)	4 (0;0;10)	2 (0;0;4)	+	-	-
	
	prostate only	89 (79;100;100)	9	13 (1;9;24)	4 (-5;0;11)	-3 (-11;0;4)	+	-	-

sexual function score	prostate with SIB	35 (15;37;51)	12	7 (0;3;15)	7 (0;7;17)	5 (-3;5;18)	+	+	-
	
	prostate only	34 (11;30;54)	12	6 (0;9;14)	4 (-3;5;12)	11 (0;7;23)	-	-	+

sexual bother score	prostate with SIB	51 (25;31;94)	18	7 (-2;3;20)	6 (0;6;19)	14 (0;0;28)	-	-	+
	
	prostate only	57 (20;44;100)	19	4 (0;0;19)	-6 (-11;0;17)	8 (0;9;27)	-	-	-

Abbreviations: SD = standard deviation of baseline value

Only sexual scores decreased significantly (> 5 points; all mean changes < 0.5 standard deviation of baseline value) at time D. As sexual scores demonstrate the largest variability (Table 3), a possible decrease strongly depends on the baseline scores. Sexual function scores of patients with a baseline sexual function score < 25 did not change > 5 points at time D in comparison to baseline in both subgroups. However, sexual function scores of patients with a baseline sexual function scores of 25-50 and > 50 decreased 12 points in both subgroups and 11/15 points in the subgroup treated with/without SIB, respectively. Considering only patients with higher baseline scores (> 25 points), changes more than a year after the end of radiotherapy also exceeded the 0.5 of standard deviation threshold (p < 0.01 and p = 0.02 with and without SIB). At time D, 75% and 60% of patients in the prostate with SIB and prostate only groups who reported erections sufficient for sexual intercourse before treatment retained this ability.

The rates of reporting big or moderate bother with specific symptoms are shown in Table 4. In accordance with the corresponding scores, the greatest changes were reported in the acute phase at the end of treatment for irritative symptoms - or urinary and bowel function overall. Significant differences between the subgroups with or without SIB have not been found. Remarkably, not a single patient reported big or moderate bother with bloody stools at times C or D. Mean bleeding function scores were 98 points at all response intervals (times A, B, C and D) in the subgroup with SIB.

**Table 4:** Rates of reporting big or moderate bother with specific symptoms

		time A	time B	time C	time D
dripping or leaking urine	prostate with SIB	7%	11%	5%	2%
	
	prostate only	10%	6%	14%	15%

need to urinate frequently	prostate with SIB	22%	46%	26%	16%
	
	prostate only	19%	50%	38%	25%

urinary function overall	prostate with SIB	9%	38%	18%	9%
	
	prostate only	24%	38%	33%	15%

urgency to have a bowel movement	prostate with SIB	7%	19%	12%	10%
	
	prostate only	14%	18%	14%	10%

losing control of stools	prostate with SIB	0%	3%	5%	7%
	
	prostate only	0%	6%	10%	0%

bloody stools	prostate with SIB	0%	0%	0%	0%
	
	prostate only	0%	6%	0%	0%

bowel habits overall	prostate with SIB	4%	13%	14%	12%
	
	prostate only	10%	25%	14%	10%

sexual function overall	prostate with SIB	49%	46%	52%	68%
	
	prostate only	50%	31%	41%	53%

GTV_PET _volumes and distances to the bladder and rectum wall were evaluated in the subgroup of patients who were treated with SIB (Table 5; < or ≥median, respectively). A tendency for larger QoL changes was found for patients with larger GTV_PET _volumes and closer distances to the bladder and rectum wall. In contrast to a faster return to baseline levels at time C of patients with GTV_PET _< 4 cm^3^, a distance to the bladder ≥1 cm and a distance to the rectum wall ≥3 mm, scores of patients with GTV_PET _> 4 cm^3 ^(bowel bother) a distance to the bladder < 1 cm (urinary bother) and a distance to the rectum wall < 3 mm (bowel bother) were still significantly higher at time C in comparison to baseline levels. The difference exceeded 5 points and the 0.5 standard deviation of baseline level threshold with the exception urinary bother scores in case of a GTV_PET _> 4 cm^3^. No statistically significant differences relative to baseline levels were found in any subgroup at time D.

**Table 5:** Mean bother scores before treatment and differences after treatment relative to baseline scores (quartiles in brackets; negative differences indicate a quality of life improvement; bold numbers indicate significant difference between treatment groups)

		time A	0.5 SD	time A-time B	time A-time C	time A-time D	significant differences		
							
							A vs. B	A vs. C	A vs. D
urinary bother score	GTV_PET _< 4 cc	81 (75;84;92)	7	17 (-5;23;32)	0 (-11;-4;11)	1 (-11;-4;7)	+	-	-
	
	GTV_PET _≥4 cc	83 (75;86;98)	8	15 (0;7;34)	5 (-9;0;14)	-2 (-9;0;4)	+	-	-

urinary bother score	distance GTV_PET _to bladder ≥1 cm	81 (75;86;90)	6	13 (2;14;37)	0 (-4;4;20)	-3 (-11;0;0)	+	-	-
	
	distance GTV_PET _to bladder < 1 cm	81 (75;82;96)	9	21 (0;21;37)	8 (-4;0;21)	4 (-7;0;11)	+	+	-

bowel bother score	GTV_PET _< 4 cc	89 (86;94;100)	8	10 (-4;14;27)	**-1** **(-7;0;4)**	-1 (-7;0;4)	+	-	-
	
	GTV_PET _≥4 cc	95 (89;100;100)	5	15 (1;12;21)	**9** **(0;2;14)**	3 (0;0;4)	+	+	-

bowel bother score	distance GTV_PET _to rectum ≥3 mm	91 (89;96;100)	7	11 (0;12;19)	2 (-6;0;4)	-1 (0;0;4)	+	-	-
	
	distance GTV_PET _to rectum < 3 mm	91 (88;98;100)	7	14 (-6;14;27)	7 (0;4;11)	2 (0;0;5)	+	+	-

Abbreviations: SD = standard deviation of baseline value

## Discussion

The whole prostate is the standard target volume for external beam radiotherapy and brachytherapy for localised prostate cancer, as prostate cancer is known to be a multifocal disease and diagnosis of microscopic cancer is not possible with presently available imaging modalities [5]. However, advances in imaging and radiotherapy techniques offer innovative treatment possibilities [15]. Treatment planning with ^18^F-choline PET-CT allows a dose escalation to a macroscopic intraprostatic lesion without increasing toxicity.

Magnetic resonance imaging (MRI), magnetic resonance spectroscopy (MRS) and choline PET/CT are suitable methods to localize intraprostatic lesions with an adequate sensitivity and specificity [6,7,16,17]. Choline acts as a precursor for the biosynthesis of phospholipids, e.g. phosphatidylcholine, the major component of the plasma membrane. The uptake of choline reflects both proliferation and signaling in transformed cells [18]. Elevated choline to citrate ratios are used to discriminate prostate cancer in MRS imaging [16]. Disadvantages of MRS in comparison to PET imaging are larger voxel sizes (usually at least 1 cm^3 ^vs. 4 mm^3^) and a more strenuous and subjective analysis of the data.

Image-guided radiotherapy (IGRT) and IMRT are prerequisites for applying the simultaneous integrated boost concept. IGRT techniques eliminate inaccuracies due to interfractional prostate displacement and help to minimize safety margins considerably. IMRT brings the opportunity to deliver heterogeneous dose prescriptions within the target. The simultaneous integrated boost concept has been rapidly adopted for the IMRT of head and neck cancer patients [19]. Dose escalation in the area of the primary macroscopic tumour is also applied in several other regions, probably most frequently for breast cancer - the clinical benefit on local control has been demonstrated [20].

In a recently published study, an intraprostatic lesion could be identified in 65 of 66 consecutive patients after performing ^18^F-choline PET-CT [21]. GTV_PET _and SUV levels were found to depend on prognostic risk factors, particularly a Gleason score > 7. Increasing PET-defined tumor volumes and increasing SUV levels correlated well with increasing prognostic risk. Though antiandrogen hormonal therapy is known to decrease choline uptake in prostate cancer [19], the identification of a lesion is still possible after NHT. SUV levels in intraprostatic lesions were comparable to patients without NHT. NHT or a prior radiation dose to the prostate reduces cell metabolism and absolute SUV both in the tumour and the benign prostate tissue [22]. A relative SUV threshold seems to be a more reasonable definition for these patients. The ratio between tumour and normal tissue is the key to identifying aggressive disease [23].

Treatment planning studies evaluating the impact of the simultaneous integrated boost have been already published, demonstrating the potential of a considerable dose escalation to the macroscopically defined tumor without significantly increasing the dose to the organs at risk or NTCP [8,24]. These results are supported by the treatment plan-related data of the patients in this study. EUD and NTCP values for the rectum and bladder have not been found to differ significantly between patients treated with or without SIB.

Quality of life evaluations after radiotherapy for prostate cancer with a SIB have not been published before. The results of this clinical study well support the results of prior treatment planning studies [8,24]. The additional SIB did not increase QoL changes in the acute phase or more than one year after radiotherapy. Generally, the treatment was very well tolerated. Even with larger SIB volumes and close distances to the bladder and/or rectum walls, mean long-term urinary and bladder QoL scores did not decrease more than 5 points (or 0.5 standard deviation) in comparison to the baseline level. Treatment tolerance did not decrease in comparison to our patients who were treated up to total doses of 70.2-72Gy using three-dimensional conformal planning without IGRT [2,3] (data presented in prior publications). In particular, rectal bleeding - usually in focus of rectal toxicity after radiotherapy for prostate cancer [2,3]- has not been found to be considerably relevant for the patients. A clinically relevant QoL change was only found in the sexual domain for patients with higher baseline sexual function scores, without a significant difference with vs. without SIB.

Acute toxicity results up to three months after RT with an MRI/MRS detected SIB have been published by Fonteyne et al. [16]. An intraprostatic lesion was identified in only 118 of 230 patients - a considerably lower percentage in comparison to treatment planning after ^18^F-choline PET-CT. The severity and incidence of acute toxicity did not increase for patients treated with SIB. However, the authors did not consider any margin around the intraprostatic lesion.

## Conclusions

According to the results of this prospective non-randomized study, treatment planning with ^18^F-choline PET-CT allows a dose escalation to a macroscopic intraprostatic lesion without increasing toxicity. Previously found significantly decreasing long-term bowel scores (> 5 points) after conformal RT to doses of 70.2-72Gy could be avoided after IMRT without and with SIB in spite of increasingly higher total doses to the prostate and specifically the intraprostatic malignant lesion.

The results are encouraging to continue evaluating this concept in the future. Further results with longer follow-up and larger patient groups are needed.

## Competing interests

The authors declare that they have no competing interests.

## Authors' contributions

MP, MJE have made substantial contributions to conception and design; MP, MDP, RH, JK, VD, NEC, MC, OW, TK, FMM, MJE have made substantial contributions to acquisition of data; MP, MDP, RH, MJE to analysis and interpretation of data. MP has been involved in drafting the manuscript. MDP, RH, JK, VD, NEC, MC, OW, TK, FMM, MJE revised it critically for important intellectual content. All authors have given final approval of the version to be published.
